# Characterization and Stability of Tanshinone IIA Solid Dispersions with Hydroxyapatite

**DOI:** 10.3390/ma6030805

**Published:** 2013-03-06

**Authors:** Xiaopan Wang, Li Li, Wei Huo, Lulu Hou, Zhiying Zhao, Weiguang Li

**Affiliations:** 1 State Key Laboratory of Natural Medicines, China Pharmaceutical University, No. 24 tongjiaxiang, Nanjing 210009, China; E-Mails: wangxiaopancpu@163.com (X.W.); whuo2009@gmail.com (W.H.); hoululu_12345@hotmail.com (L.H.); 2 Department of Pharmacy, the Second Affiliated Hospital, Nanjing Medical University, No. 121 jiangjiayuan, Nanjing 210028, China; E-Mail: lilipharm@yeah.net

**Keywords:** TanIIA, hydroxyapatite, solid dispersions

## Abstract

Solid dispersions of tanshinone IIA (TanIIA) using hydroxyapatite (HAp) as the dispersing carrier (TanIIA-HAp SDs) were prepared by the solvent evaporation method. The formed solid dispersions were characterized by scanning electron microscopy (SEM), differential scanning calorimetry analysis (DSC), X-ray powder diffraction (XRPD) and Fourier transforms infrared (FTIR) spectroscopy. The* in vitro* dissolution rate and the stability of TanIIA-HAp SDs were also evaluated. DSC and XRPD showed that TanIIA was changed from a crystalline to an amorphous form. FTIR suggested the presence of interactions between TanIIA and HAp in solid dispersions. The result of an* in vitro* dissolution study showed that the dissolution rate of TanIIA-HAp SDs was nearly 7.11-folds faster than free TanIIA. Data from stability studies for over one year of TanIIA-HAp SDs performed under room temperature revealed no significant differences in drug content and dissolution behavior. All these results indicated that HAp may be a promising carrier for improving the oral absorption of TanIIA.

## 1. Introduction

Tanshinone IIA (TanIIA), a highly lipophilic compound, isolated from the Chinese medicinal herb *Salvia miltiorrhiza*, has been reported to treat cardiovascular and cerebrovascular diseases, including angina pectoris, arrhythmias, acute ischemic stroke and hyperlipidemia [1,2,3,4,5,6]. However, it was limited in clinical use, due to its poor oral absorption (below 3.5%) caused by its poor solubility and low dissolution rate [7,8,9,10,11,12]. Therefore, a pharmaceutical strategy for promoting its solubility or dissolution rate should be designed to develop tanshinone IIA as a new drug candidate. 

Solid dispersion is one of the most successful techniques, which could enhance the dissolution rate of poorly aqueous soluble drugs by particle size reduction, amorphous fraction and wettability improvement [13,14,15]. The high energy state of the amorphous form confers higher solubility and improves bioavailability through increasing dissolution. Thus, the application of amorphous phases has been the subject of very intensive investigations in the pharmaceutical field [16,17,18]. However, the thermodynamic instability of this amorphous state is generally associated with the high energy state and may lead to unacceptable physical changes, such as recrystallization during storage [19,20]. Although factors governing physical stability remain controversial and complicated, it was well known that specific interactions between drug and carrier may retard this conversion to the crystalline form [21,22,23]. As reported by Wu *et* *al*. [24] and Al-Obaidi *et al*. [25], the hydrogen bonding between drug and carrier is responsible for drug dispersion and depresses the crystallization of drug in solid dispersions.

Hydroxyapatite (HAp) is a natural element of human hard tissues (70% of bone is made up of this organic mineral) and has been commonly studied in bone tissue engineering, owing to its good biocompatibility [26,27,28,29]. HAp is known for its bioactive (*i.e.*, ability of forming a direct chemical bond with surrounding tissues), osteoconductive, non-toxic, non-inflammatory and non-immunogenic properties [30,31]. Furthermore HAp has been applied widely in various biomedical applications, owning to its unique functional properties of high surface-area-to-volume ratio and porosity. Its ultrafine structure, which is similar to biological apatite’s, had a great impact on cell-biomaterial interaction [32,33,34]. However, HAp has not yet been reported as a carrier of solid dispersions. Therefore, TanIIA-HAp SDs were prepared by the solvent evaporation method; and they were studied by scanning electron microscopy (SEM), differential scanning calorimetry analysis (DSC), X-ray powder diffraction (XRPD) and Fourier transforms infrared (FTIR) spectroscopy, along with the* in vitro* dissolution rate and stability. Through this research, we expect to pave the preliminary way towards the feasibility of HAp as a carrier of solid dispersions. 

## 2. Experimental Section 

### 2.1. Materials 

TanIIA with 98% purity was purchased from the Nanjing ZeLang Medical Technology Co., Ltd. TanIIA standards were purchased from the National Institute for the Control of Pharmaceutical and Biological Products (Beijing, China). HAp was supplied by Shanghai Jiang Lai Bio-Technology Co., Ltd. All reagents were of analytical grade, except methanol, which was of chromatographic grade.

### 2.2. Preparation of Solid Dispersions and Physical Mixtures

Solid dispersions of TanIIA with HAp in various weight ratios were prepared using the solvent evaporation method. In brief, TanIIA was dissolved in ethanol and HAp was suspended into the above solution (the weight ratio of TanIIA and HAp was 1:3, 1:5, 1:7 and 1:9). The formed suspension was evaporated under reduced pressure in a rotavapor (Buchi, Switzerland) at 45 °C. The obtained solid dispersions were further dried in a vacuum chamber (Heraeus, Germany) at room temperature for 12 h to remove the remaining ethanol. Subsequently, samples were stored in a desiccator until further analysis. Physical mixtures were prepared by mixing TanIIA with HAp, then grinding thoroughly with a mortar and pestle until homogeneous mixtures were obtained.

### 2.3. *In vitro* Dissolution Study

#### 2.3.1. HPLC Analysis of TanIIA

The concentration of TanIIA in the dissolution medium was determined by using a high performance liquid chromatography (HPLC) system (Shimadzu Scientific Instrument, MD, USA), consisting of a UV detector (SPD-10A), a pump (LC-10AD) and an automatic injector (SIL-10A). The mobile phase of methanol and water (85:15, v:v) was used at a flow rate of 1.0 mL min^−1^. The samples were analyzed at 270 nm and the 30 °C temperature of a Diamonsil^TM^ RP-C18 column (250 mm × 4.6 mm, 5 μm).

#### 2.3.2. *In vitro* Dissolution Studies 

The dissolution studies were performed using the paddle method, according to the 34th edition of US Pharmacopoeia. A ZRS-8G dissolution tester (Tianjin, China) was used with 900 mL dissolution media (distilled water contained 0.5% sodium dodecyl sulfate) volume at 37 ± 1 °C and a stirring rate of 50 rpm. The samples equivalent to 5 mg TanIIA were sealed in hard gelatin capsules with a manual capsule filling machine (CapsulCN-50, Zhejiang, China) and put into the dissolution cup. At predetermined time intervals of 15, 30, 60, 90, 120 and 180 min, 5 mL of dissolution medium were withdrawn and replaced with the same medium volume. The withdrawn samples were filtrated (0.45 μm), then spectrophotometrically assayed at 270 nm. Experiments were performed in triplicate.

### 2.4. Differential Scanning Calorimetry (DSC)

The DSC profiles of TanIIA, HAp, physical mixtures and solid dispersions were obtained by using differential scanning calorimeter (204A/G Phoenix^® ^instrument, Netzsch, Germany) at a heating rate of 10 °C/min from 25 to 500 °C in a nitrogen atmosphere.

### 2.5. Scanning Electron Microscopy (SEM)

SEM samples were mounted on aluminum stubs and coated with a thin gold–palladium layer using an auto-fine coater unit (Jeol, JFC, Tokyo, Japan). The surface topography was analyzed with a Jeol scanning electron microscope (JSM-6360A, Tokya, Japan) operated at an acceleration voltage of 30 kV.

### 2.6. X-ray Powder Diffraction (XRPD)

XRPD was performed at room temperature with a X-ray diffractometer (X-pro Pan analytical, Phillips, Mumbai, India). The data were collected through primary monochromated radiation (Cu Ká1, ë =1.5406 Å), over a 2θ range of 0°–70° with a step size of 0.04 and a dwell time of 10 s per step. About 200 mg of each sample powder were side-loaded in a sample holder to minimize possible preferential orientation. 

### 2.7. Fourier Transform Infrared Spectroscopy (FTIR)

FTIR spectroscopic analysis was carried out using a Nicolet Nextus 470 FTIR spectrometer (Thermo Electron Corporation, USA). The infrared spectra of the samples were recorded in the solid state using the KBr disc method over a wave number range of 4000–400 cm^−1^. Individual HAp, TanIIA and physical mixtures were run as controls.

### 2.8. Stability Test

The prepared solid dispersions were stored for 12 months at 25 ± 2 °C and 60% ± 5% relative humidity in an artificial climate box. The extent of dissolution was analyzed at predetermined time intervals of 0, 3, 6, 9, 12 months. 

## 3. Results and Discussion

### 3.1. *In vitro* Dissolution Study

**Figure 1:**
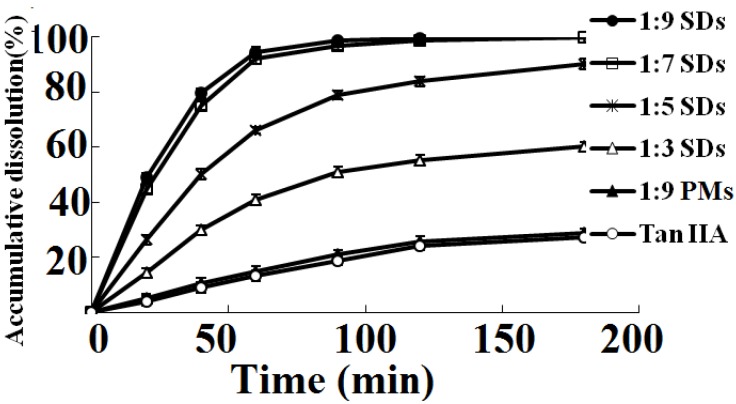
The dissolution profiles of tanshinone IIA (TanIIA) and solid dispersions (SDs) at different TanIIA/HAp (hydroxyapatite) ratios of 1:3, 1:5, 1:7, 1:9 and the 1:9 physical mixtures. Each point represents the mean ± SD (*n* = 3).

The dissolution rate of TanIIA from different samples is provided in Figure 1. TanIIA crystals exhibited a low dissolution rate, with a 13.3% release in 60 min and only reaching 27.3% in 180 min, while the dissolution rate of TanIIA from the physical mixture had no significant difference compared with the free TanIIA. Furthermore, as we expected, an apparent trend was observed between the HAp ratios and the dissolution rate of TanIIA. The 1:9 SDs released 94.6% of drug in 60min, whereas 1:3, 1:5 and 1:7 SDs exhibited a release of 40.9%, 66.3% and 92.3%, respectively. Moreover, 1:7 and 1:9 SDs showed similar release curves and higher dissolution rates than the 1:3 and 1:5 SDs. These indicated that 7–9 folds of HAp carrier was enough for TanIIA SDs. In view of the need of long-term stability for SDs, 1:9 ratio SDs were chosen for further study. 

There were several factors explaining the improved drug dissolution from solid dispersions, such as the enhancement in wettability and dispersibility of TanIIA, as well as the decrease in particle size. The presence of amorphous TanIIA might be a significant factor, which was confirmed by the results obtained from SEM, DSC and XRPD. HAp is a good adsorbent for a wide range of ions, small molecules and macromolecules, owing to its unique functional properties of high surface-area-to-volume ratio [35,36]. Additionally, adsorption onto insoluble, porous, high surface-area carriers is a well-known technique to enhance drug dissolution and has already been described for silica-based excipients in the early 1970s [37]. 

### 3.2. Scanning Electron Microscopy (SEM)

**Figure 2:**
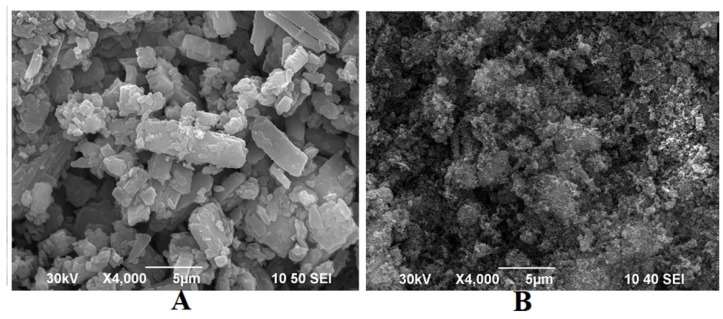
Scanning electron microscopy (SEM) photomicrographs of TanIIA (**A**) and 1:9 (w/w) SDs (**B**).

Figure 2 shows the micrographs for the pure TanIIA and solid dispersions (1:9 SDs). The particle size of TanIIA (Figure 2A) was in the range of 1–5 μm, with a prism-like crystal structure. In contrast, no crystal structure of the drug existed in SDs (Figure 2B), indicating the transformation of TanIIA to an amorphous state, which conforms to the results of the dissolution test.

### 3.3. Differential Scanning Calorimetry (DSC)

**Figure 3:**
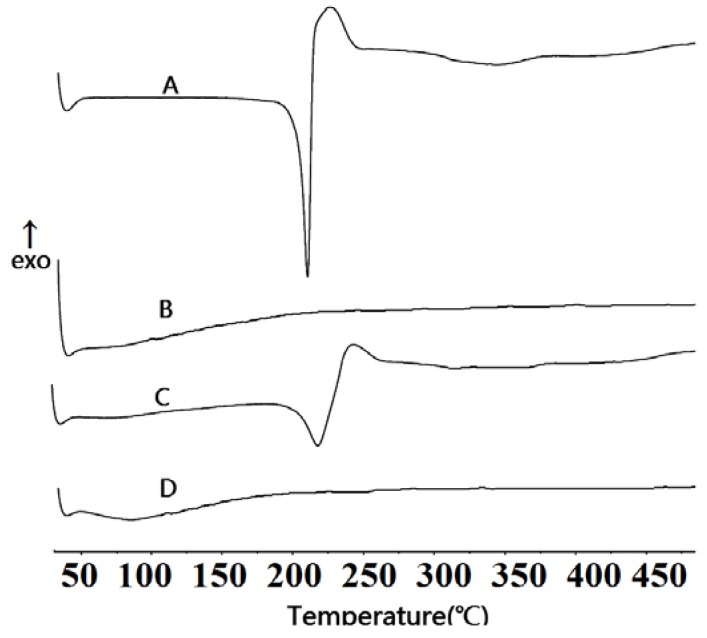
Differential scanning calorimetry (DSC) curves of TanIIA (**A**); hydroxyapatite (HAp) (**B**); 1:9 (w/w) physical mixtures (**C**) and 1:9 (w/w) SDs (**D**).

DSC thermograms of samples are shown in Figure 3. The thermogram of pure TanIIA (Figure 3A) showed typical characteristics of a crystalline product, with a single endothermic process at about 209 °C, related to its melting point [34], as well as with a subsequent exothermic process caused by the thermal degradation of the TanIIA at 224.59 °C. For physical mixtures (Figure 3C), the melting and exothermic peak of TanIIA was also observed, demonstrating that the drug appeared in a crystal form. As shown in Figure 3D, the complete disappearance of the endothermic peak corresponding to TanIIA was observed in solid dispersions, which gave supporting evidence for the presence of TanIIA’s amorphous state [38].

### 3.4. X-ray Powder Diffraction (XRPD)

**Figure 4:**
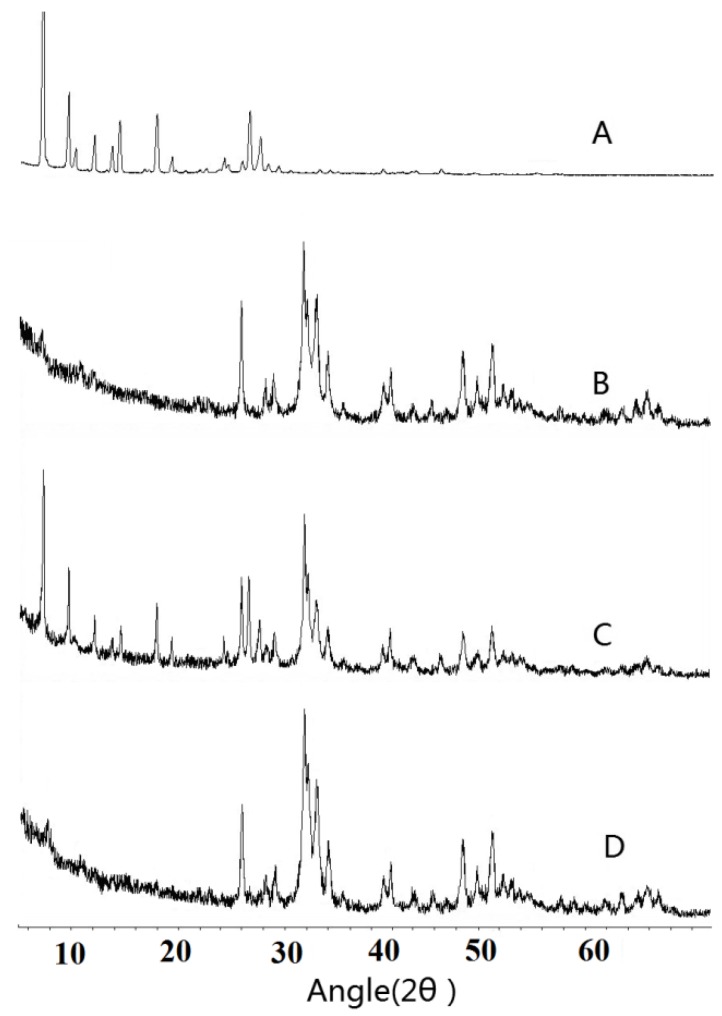
The X-ray powder diffractograms: TanIIA (**A**); HAp (**B**); 1:9 (w/w) physical mixtures (**C**) and 1:9 (w/w) SDs (**D**).

The X-ray powder diffraction of TanIIA, HAp, physical mixtures and corresponding SDs are shown in Figure 4. Pure TanIIA (Figure 4A) had sharp and intense peaks in the range of 5°–45° at 2θ angles, which suggested the presence of the crystalline from of TanIIA. HAp (Figure 4B) showed some characteristic strong 2θ peaks between 25° and 65°. In physical mixtures (Figure 4C), both the characteristic diffraction peaks of the carrier and drug were present. By contrast, XRPD patterns of SDs (Figure 4D) showed the absence of any trace of the crystallinity of TanIIA, indicating the amorphous state of TanIIA. These results also suggested that the drug was transformed into amorphous forms during solid dispersing process, which is in parallel with the DSC thermograms.

### 3.5. Fourier Transform Infrared (FTIR) Spectrophotometry

**Figure 5:**
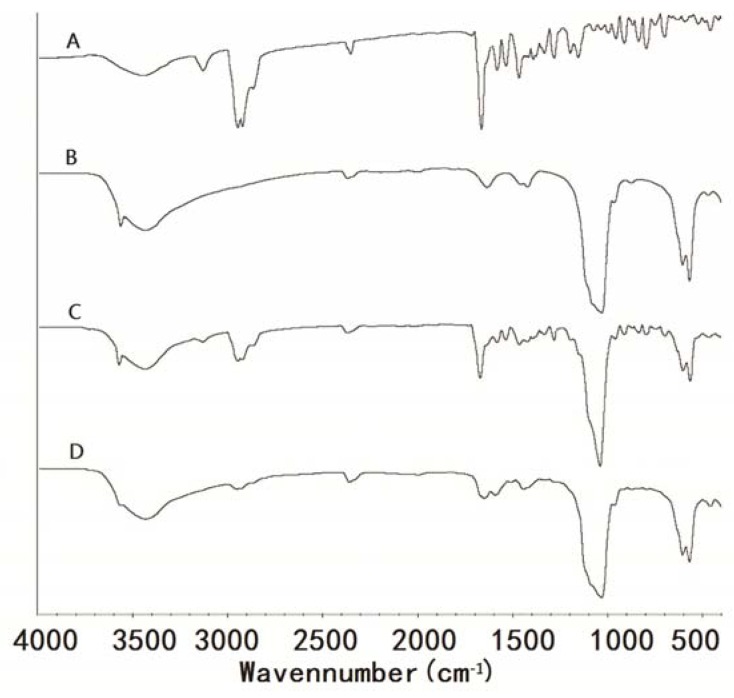
Fourier transform infrared (FTIR) spectra of TanIIA (**A**); HAp (**B**); 1:9 physical mixtures (**C**) and 1:9 SDs (**D**).

Fourier transform infrared spectrophotometry (FTIR) is a useful tool for identifying the interactions between drug and excipient, due to its surface scan properties. Figure 5A illustrates the spectrum of pure TanIIA, with strong absorption peaks at 1678 cm^−1^, which is attributed to the carbonyl-stretching vibration [39]. Figure 5B shows the peaks of HAp at 3577 cm^−1^, which corresponded to the –OH stretching vibration mode; the peaks at 1053 cm^−1^, 605 cm^−1^ and 576 cm^−1^ indicated the presence of phosphate groups, which have been proven by Shaltout *et al.* [40] and Hwang *et al.* [41]. The FTIR spectrum of physical mixtures (Figure 5C) was almost equivalent to the spectrum of TanIIA and HAp separately, which suggested that there was no chemical interaction between HAp and TanIIA in physical mixtures. However, from the spectrum of SDs (Figure 5D), it was shown that the weak –OH stretching vibration peak was observed at 3577 cm^−1^ and the lower frequencies of the carbonyl-stretching vibration peak were shifted from 1678 cm^−1^ to 1655 cm^−1^, which suggested that TanIIA interacted with HAp, presumably by hydrogen bonds. Hydrogen bonding formation probably improved the wettability properties of the drug and further increased the drug dissolution rate.

### 3.6. Stability Test

**Figure 6:**
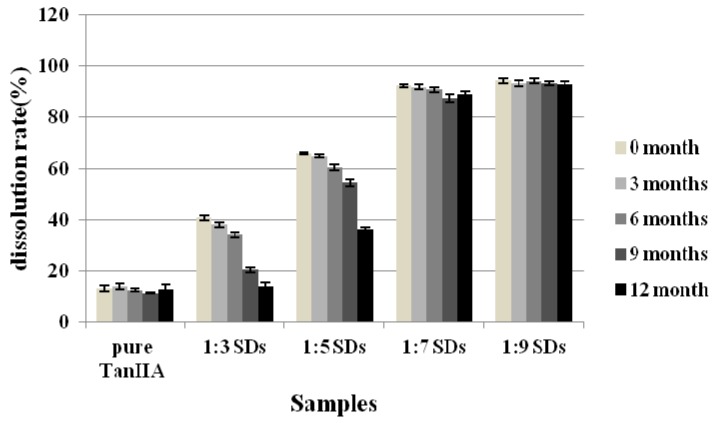
The dissolution rate of TanIIA in 1 h from different samples.

The long-term stability of the TanIIA-HAp SDs was also investigated, and the results are shown in Figure 6. After 12 months, there was no considerable difference with regard to the dissolution rate for three samples of pure drug, 1:7 SDs and 1:9 SDs, which suggested that TanIIA still maintained the amorphous form in those two ratio SDs. *Vice versa*, 1:3 and 1:5 SDs showed a significant reduction of the dissolution rate of 1 h by 36.3% for 1:3 SDs, while it was 13.9% for 1:5 SDs. This leads to the conclusion that a specific ratio between TanIIA and HAp should be obtained to have stable solid dispersions. 

Furthermore, the improved stability of solid dispersions might be attributed to a specific drug-carrier interaction, such as hydrogen bonding formation, which was extensively described in the literature [42,43]. As shown in the FTIR spectra, the TanIIA carbonyl group formed a hydrogen bond with the hydroxyl group of HAp in the solid dispersions, which could explain the extended stability of TanIIA. Dispersing the drug in a carrier also contributed to improved storage stability [44]. These obtained results confirmed that HAp had a strong stabilizing effect on the amorphous TanIIA in solid dispersions at room temperature. Therefore, as a novel and promising carrier of solid dispersions, Hap is worthy of further study.

## 4. Conclusions 

In this study, TanIIA solid dispersions with HAp were successfully prepared and characterized. the dissolution rate of TanIIA was significantly increased by solid dispersion technology. Data from the stability study revealed that no considerable difference of drug content and dissolution behavior for over one year was observed in an optimized formula of SDs. Thus, the present research indicates the feasibility of using HAp as a potential carrier of SDs to improve the solubility and the dissolution rate of insoluble drug and, hence, to enhance its oral absorption. It is worth pointing out that further research is needed to investigate HAp’s toxicity or biocompatibility for oral administration in view of a few reports dedicated to its oral drug delivery. 
